# A systematic review of the cost-effectiveness of interventions to increase cervical cancer screening among underserved women in Europe

**DOI:** 10.1007/s10198-023-01627-1

**Published:** 2023-09-20

**Authors:** Li Sun, Shruti Patel, Camilla Fiorina, Audrey Glass, Lise Rochaix, The CBIG-SCREEN Consortium, Marc Bardou, Berit Andersen, Pia Kirkegaard, Rikke Buus Bøje, Mette Tranberg, Martin McKee, Sadie Bell, Rachel Greenley, Samuel Rigby, Paolo Giorgi Rossi, Luca Ghirottoo, Letizia Bartolini, Noemi Auzzi, Paola Mantellini, Giusy Iorio, Laura Bonvicini, Nuno Lunet, João Firmino-Machado, Margarida Teixeira, Ana Fernandes, Mariana Amorim, Inês Baía, Romeu Mendes, Cláudia Gouvinhas, Anneli Uusküla, Anna Tisler, Aadriana Baban, Diana Tăut, Nicoleta Jiboc, Florian Nicula, Alexandra Tolnai, Rebecca Moore, Vanessa Moore, Partha Basu, Isabel Mosquera Metcalfe, Keitly Mensah, Eric Lucas, Irina Todorova, Yulia Panayotova, Tatyana Kotzeva, David Ritchie, Helena Ros Comesana, Meritxel Mallafré-Larrosa, Ginevra Papi, Christiane Dascher-Nadel, Anna M. Foss, Rosa Legood

**Affiliations:** 1 Department of Health Services Research and Policy, Faculty of Public Health and Policy, London School of Hygiene and Tropical Medicine, London, UK; 2 Paris School of Economics, Paris, France; 3 Department of Global Health and Development, Faculty of Public Health and Policy, London School of Hygiene and Tropical Medicine, London, UK

**Keywords:** Cervical cancer screening, Cost-effectiveness, Uptake rates, Coverage, Attendance, Inequalitie

## Abstract

**Background:**

This study aimed to conduct a systematic review of the cost-effectiveness studies of interventions to increase cervical cancer screening uptake rates in underserved women in Europe.

**Methods:**

A search of Embase, Medline, Global Health, PsychINFO, and NHS Economic Evaluation Database was conducted for studies published between January 2000 and September 2022. Studies were eligible if they analysed the cost-effectiveness of any interventions to improve participation in cervical cancer screening among underserved women of any age eligible to participate in cervical cancer screening in European countries, in any language. Study characteristics and cost-effectiveness results were summarised. Study quality was assessed using the Drummond Checklist, and methodological choices were further compared.

**Results:**

The searches yielded 962 unique studies, with 17 of these (from twelve European countries) meeting the eligibility criteria for data extraction. All studies focused on underscreened women as an overarching group, with no identified studies focusing on specific subgroups of underserved women. Generally, self-HPV testing and reminder interventions were shown to be cost-effective to increase the uptake rates. There was also research showing that addressing access issues and adopting different screening modalities could be economically attractive in some settings, but the current evidence is insufficient due to the limited number of studies.

**Conclusion:**

This systematic review has revealed a gap in the literature on the cost-effectiveness of interventions to improve uptake rates of cervical cancer screening through tailored provision for specific groups of underserved women.

**Supplementary Information:**

The online version contains supplementary material available at 10.1007/s10198-023-01627-1.

## Background

Cervical cancer is the fourth most common cancer and the second leading cause of cancer deaths among women^1^ worldwide [1]. In Europe, over 61,000 women are diagnosed with cervical cancer and nearly 26,000 women die from the disease every year [2]. Cervical cancer can largely be prevented with either vaccination against high risk Human Papilloma Virus (hrHPV) or screening of those with HPV infection and treatment of pre-cancerous lesions [3]. Most cervical cancer deaths that occur in Europe today can be largely attributed to unvaccinated women with low cervical cancer screening rates, disproportionately concentrated in women with a variety of characteristics that render them vulnerable [4–7].

Worryingly, uptake of cervical cancer screening is highly variable, both between and within countries. Globally, a decreasing proportion of eligible women are being screened during the past decade [8]. In Europe, rates vary between 25 and 80% and, even in countries such as the UK with historically high screening rates, uptake has been falling in recent years to 71% in 2019, despite previously being over 80% [9]. This is a concern because there is now considerable evidence that those women who have not been vaccinated against HPV are less likely to be screened, leading to widening inequalities [10–13].

Despite the well-known socio-economic gradient in cervical cancer morbidity and mortality, existing screening programmes efforts underserve women from disadvantaged and marginalised groups, including the poor, those from certain ethnic minorities, incarcerated women, LGBTQ + women, transgender women, sex workers, and migrants [6, 14–16]. Women with comorbidities such as HIV, mental illness, alcohol or substance misuse, and disabilities are also underscreened [17]. It is important to note that women may belong to one or more of these underserved groups and that risk factors can interact. These women often have a higher background risk of cervical cancer either due to higher prevalence of hrHPV or increased vulnerability to HPV infection due to existing health inequalities or comorbidities (notably HIV) [15]. Barriers experienced when seeking access to healthcare by minoritised groups also reduce screening uptake [18]. This strong socio-economic gradient in screening participation, as well as the disproportionate representation of marginalised groups amongst unvaccinated women has created significant inequalities in the prevention of cervical cancer [19].

There are a number of interventions that have been proposed to increase cervical cancer screening uptake rates among underserved populations, such as screening reminders, HPV self-sampling, removal of financial barriers, and educational interventions. This paper reviews cost-effectiveness studies of interventions to increase cervical cancer screening uptake rates in underserved women in Europe.

## Methods

This study has been registered in PROSPERO (CRD42022310195) and an ethical exemption has been granted by LSHTM since it is a literature review (reference 25260).

### Eligibility criteria

Underserved women were identified based on existing literature and include women who are vulnerable by virtue of socio-economic disadvantage, unvaccinated against hrHPV, underscreened, from minority sexualities or gender identity groups (LGBTQI + including trans men and women), from minority ethnic groups, disabled, migrants, sex workers, incarcerated, living with HIV or other STIs, living with mental illness, or living with addiction disorders.

The inclusion criteria were based on the PICOS framework: (i) population: underserved women of any age eligible to participate in cervical cancer screening in European countries; (ii) intervention: any intervention(s) to improve participation in cervical cancer screening; (iii) comparator: standard practice or no screening; (iv) outcome: cost-effectiveness measures; and (v) study design: economic evaluations.

We excluded studies with the following characteristics: (i) evaluating the cost-effectiveness of different screening tests (e.g. cytology or HPV testing) rather than interventions to improve cervical cancer screening uptake rates; (ii) review articles; (iii) studies published before January 2000; and (iv) earlier publications of studies with results that have been well captured in subsequent studies.

### Search methods

In September 2022, we searched Embase, Medline, Global Health, PsychINFO, and NHS Economic Evaluation Database with search terms in Appendix Table 1. Titles and abstracts were reviewed in a double-blinded screening approach, and any disagreements on which abstracts should be screened in or out were reconciled by discussions. Full-texts of the studies that potentially met the eligibility criteria were retrieved and reviewed.

### Data extraction and synthesis

Two investigators independently extracted the study characteristics, including settings, years since the last screen for non-attenders, interventions, comparators, outcome types, incremental costs, incremental health outcomes, incremental cost-effectiveness ratios (ICERs), and conclusions. Any disagreements were resolved by discussions or a third reviewer.

Health outcomes could be measured as quality-adjusted life years (QALYs), life years gained, the number of women screened, or the number of detected high-grade cervical intraepithelial neoplasia (CIN2 +). We used the Gross Domestic Product (GDP) deflator to convert costs and ICERs to EUROs with the base year of 2020 to facilitate comparison across different healthcare settings and time points. Data were extracted into an Excel table and then written into text by way of a narrative synthesis.

### Critical appraisal and methodological assessment

The established checklist by Drummond et al. [20] was used to assess the quality of the reviewed studies. In addition, we conducted a more detailed analysis of the methods used, including the economic model types, cost analysis perspectives, time horizons, discount rates, and whether any uncertainty was explored by sensitivity analyses. The economic model types (decided a priori) could include the decision tree model, Markov model, microsimulation model, or trial-based analysis without modelling applied.

## Results

**Fig. 1:**
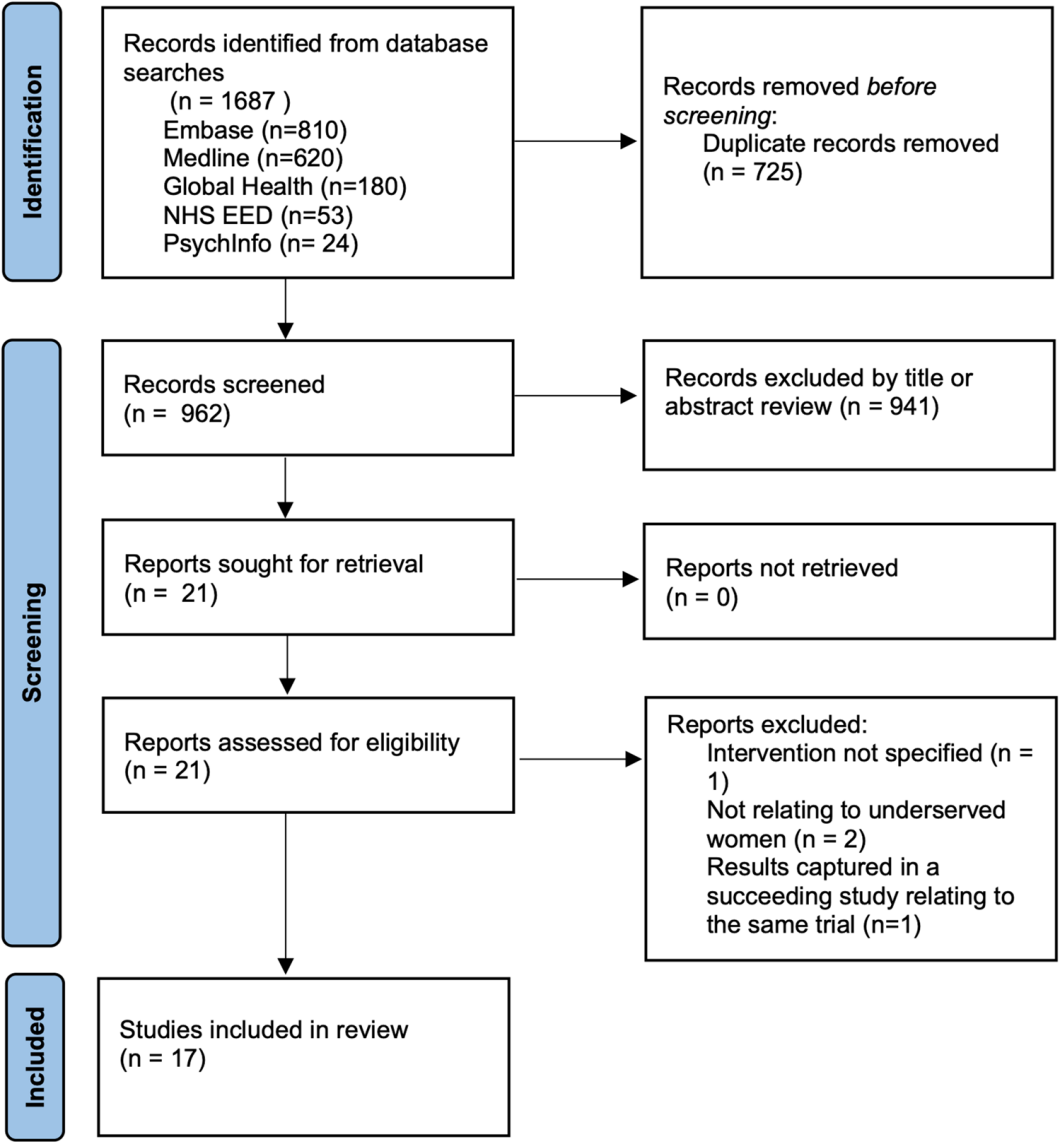
Study Flow Diagram

Embase search yielded 810 possible studies, Medline yielded 620, Global Health yielded 180, NHS Economic Evaluation Database yielded 53 and PsychINFO yielded 24. The collective searches yielded 962 unique studies after removing duplicates. Based on the eligibility criteria, we excluded 945 studies and included 17 studies in this review (Fig. 1). Table 1 summarises the study characteristics and cost-effectiveness of interventions increasing cervical cancer screening uptake rates. In accordance with SWiM guidance, the studies have been grouped to aid synthesis. Table 1 is subdivided into two sections based on study type: cost-utility analysis or cost-effectiveness analysis, and within each category, studies are grouped by intervention type: self-sampling, reminder, and programmatic intervention [21].

**Table 1:** Basic characteristics and cost-effectiveness of interventions increasing cervical cancer screening uptake rates

Study	Setting	Years since last screen for non-attenders	Intervention	Intervention uptake rate	Comparator	Outcome	Incremental costs	Incremental health outcomes	ICER (EURO 2020)	Conclusion
Cost-utility analyses										
Burger et al. [22]	Norway	≥ 8 years	Self-sampling—opt out	11–20%^t^	Standard invitation	Cost per QALY	€1242	0.047 QALY	€26,446/QALY	Cost-effective
Rozemeijer et al. [23]	Netherlands	5 years	Self-sampling—opt out	17%^t^	Standard invitation	Cost per QALY	–	–	€2377/QALY	Cost-effective
Vassilakos et al. [24]	Switzerland	3 years	1) Self-sampling (opt out) and triage with HPV 2) Self-sampling (opt out) and triage with colposcopy	1) 70%^a^ 2) 70%^a^	Standard invitation and no screening	Cost per QALY	Vs standard strategy 1) €− 464 2) Self-HIV/colpo €− 383	Vs standard strategy 1) 0.006 QALY 2) 0.008 QALY	Vs standard strategy 1) €− 75,053/QALY 2) €− 50,695/QALY	Cost-saving
Tsiachristas et al. [25]	UK	0.5 years	1) Opt in self-sampling 2) Opt out self-sampling kit 3) Nurse navigator (NN) 4) Letter with a timed appointment for a cytology 5) Letter offering women the choice of either having access to an NN or opt in self-sampling	1) 17%^t^ 2) 22%^t^ 3) 14%^t^ 4) 21%^t^ 5) 17%^t^	Standard invitation	Cost per QALY	1) €3.5 2) €38.4 3) €− 5.4 4) €25.5 5) €5.6	1) 0.0004 QALY 2) 0.0027 QALY 3) − 0.0007 QALY 4) 0.0022 QALY 5) 0.0005 QALY	1) €8421/QALY 2) €4152/QALY 3) €8221/QALY 4) €11,634/QALY 5) €10,882/QALY	1) Cost-effective 2) Cost-effective 3) Lower costs and lower outcomes 4) Cost-effective 5) Cost-effective
Firmino-Machado et al. [26]	Portugal	5 years	1) Automated short message service text messages (SMS)/phone calls/reminders 2) Automated SMS/phone calls/reminders + manual phone calls 3) Automated SMS/phone calls/reminders + manual phone calls + face-to-face interviews	1) 34%^t^ 2) 43%^t^ 3) 51%^t^ 4) 51.2%^t^	Standard invitation	Cost per QALY	Healthcare: 1) €− 1.4 2) €− 1.1 3) €0.1 Societal: 1) €− 0.7 2) €0.1 3) €1.4	1) 0.0001 QALY 2) 0.0002 QALY 3) 0.0002 QALY	Healthcare: 1) €− 11,725/QALY 2) €− 5063/QALY 3) €633/QALY Societal: 1) €− 6108/QALY 2) €553/QALY 3) € 6250/QALY	Healthcare: 1) Cost-saving 2) Cost-saving 3) Cost-effective Societal: 1) Cost-saving 2) Cost-effective 3) Cost-effective
Voko et al. [27]	Hungary	3 years	1) Communications campaign 2) Communications campaign + local delivery	1) 50%^a^ 2) 50%^a^	No screening	Cost per QALY	1) €272 2) €123	1) 0.0070 QALY 2) 0.0055 QALY	1) €39,145/QALY 2) €22,458/QALY	Cost-effective
Cost-effectiveness analyses										
Haguenoer et al. [29]	France	3 years	1) Recall letter 2) Self-sampling	1) 12%^t^ 2) 23%^t^	Standard invitation	Cost per screen	1) €4 2) €5	Incremental number of screened women: 1) 35 2) 257 (total 2000 in each group)	1) €85 per additional screen 2) €69 per additional screen	*
Bais et al. [36]	Netherlands	5 years	Self-sampling—opt out	34%^t^	Standard invitation	Cost per CIN2 +	€41	0.005 CIN2 +	€8,926 per additional CIN2 +	*
Broberg et al. [37]	Sweden	6–8 years	1) Self-sampling—opt in 2) Telephone reminder	1) 25%^t^ 2) 18%^t^	Standard invitation	Cost per CIN2 +	1) €17 2) €8	1) 0.004 CIN2 + 2) 0.002 CIN2 +	1) €4,784 per additional CIN2 + 2) €4,420 per additional CIN2 +	*
Virtanen et al. [38]	Finland	5 years	1) Primary invitation and a reminder letter 2) Primary invitation and self-sampling (opt out) 3) Two letters and self-sampling, followed by pap-smear triage 4) Two letter and self-sampling, followed by colposcopy	1) 17%^a^ 2) 32%^a^ 3) 21%^a^ 4) 21%^a^	Standard invitation	Cost per CIN2 +	1) €8 2) €5 3) €10 4) €14	1) 0.0004 CIN2 + 2) 0.0004 CIN2 + 3) 0.0008 CIN2 + 4) 0.0008 CIN2 +	1) €18,058 per additional CIN2 + 2) €11,825 per additional CIN2 + 3) €12,727 per additional CIN2 + 4) €18,192 per additional CIN2 +	*
Stein et al. [30]	UK	15 years	1) Telephone reminder 2) Invitation letter from health professional 3) Invitation letter from a celebrity	1) 1%^t^ 2) 5%^t^ 3) 2%^t^	Standard invitation	Cost per screen	1) €1001 2) €320 3) €320	Incremental number of screened women: 1) − 1 2) 8 3) 0	Letter from health professional: €40 per additional screen	*
Oscarsson et al. [31]	Sweden	5 years	Telephone reminder and practical arrangements	30%^t^	Standard invitation	Cost per screen	€8879	Incremental number of screened women: 44 (total 400 per group)	€202 per additional screen	*
Paulauskiene et al. [32]	Lithuania	3 years	1) Timed appointment letter 2) Timed appointment letter and reminder letter	1) 25%^t^ 2) 36%^t^	Standard practice (opportunistic)	Cost per screen	1) €1952 2) €4664	Proportion of additional screens: 1) 12.2% 2) 23.3%	1) €11 per additional screen 2) €15 per additional screen	*
Trapero-Bertran et al. [33]	Spain	3.5 years	1) Invitation letter 2) Invitation letter + leaflet 3) Letter + leaflet + telephone call	1) 23%^t^ 2) 19%^t^ 3) 17%^t^	Standard practice (opportunistic)	Cost per screen	1) €0.52 2) €1.95 3) €3.16	1) 17.6% 2) 16.7% 3) 21.7%	Cost per additional 1% screening coverage: 1) €3.0 2) €11.7 3) €14.6	*
Barré et al. [28]	France	3 years	Organised screening invitation and reminder letters with varying tests and frequency of screening Primary test /confirmation test after positive primary test [frequency]: 1) Pap/Pap [3 years] 2) Pap/p16Ki67 [3 years] 3) HPV/Pap [5 years] 4) HPV/Pap [3 years] 5) HPV/Pap [10 years] 6) HPV/p16Ki67 [5 years] 7) HPV/p16Ki67 [10 years] 8) HPV/p16Ki67 [3 years]	66%^t^	Standard practice (opportunistic screening using Pap/Pap or HPV [3 years])	Cost per life year	1) €23,507 2) €26,880 3) €58,820 4) €− 14,020 5) €− 77,373 6) €39,951 7) €− 68,097 8) €169,398	Additional life years: 1) 10.0 2) 11.7 3) 15.9 4) 15.9 5) 10.5 6) 18.1 7) 13.0 8) 18.4	1) €23,437/LY 2) €23,104/LY 3) €36,995/LY 4) Dominant 5) Dominant 6) €2204/LY 7) Dominant 8) €92,285/LY	*
De Jonge et al. [34]	Belgium	2.5 years	Invitation letter	76%^t^	Standard invitation	Cost per screen	€137,030	3355 (total 43,523 in intervention group)	€41	*
Diaz et al. [35]	Spain	Not specified	Organised HPV testing at 5-year intervals 1) at 40% coverage 2) at 70% coverage	40–70%^t^	Standard practice (opportunistic cytology screening at 3-year interval)	Cost per screen	Versus opportunistic at 40% coverage 1) € − 311,096 2) €3,173,796	Assuming 40% and 70% coverages	€− 9 €− 18	*

*PAP* Papanicolaou test

*The studies reported the cost per life year, per screen, or per CIN2 + as the cost-effectiveness outcomes which could not be compared with the local willingness-to-pay thresholds (cost per QALY)

^t^Uptake rate based on trial data

^a^Uptake rate based on assumptions

### Study characteristics

The identified studies were from twelve European countries: Sweden (*n* = 2), the United Kingdom (*n* = 2), the Netherlands (*n* = 2), France (*n* = 2), Spain (*n* = 2), Norway (*n* = 1), Switzerland (*n* = 1), Portugal (*n* = 1), Hungary (*n* = 1), Finland (*n* = 1), Lithuania (*n* = 1) and Belgium (*n* = 1).

All studies focused on underscreened women in all the underserved groups of interest, defined as non-attendance varying from 6 months to 15 years after invitation. No studies were identified that evaluated the cost-effectiveness of interventions to improve cervical cancer screening uptake in any other underserved groups.

Six studies were cost-utility analyses with QALYs as the health outcome [22–27], whilst the remaining 11 studies were cost-effectiveness analyses measuring health outcomes such as life years gained [28], the number of women screened [29–35], or the number of CIN2 + detected [36–38].

### Interventions and comparators

Eight studies evaluated self-HPV sampling at home [22–25, 29, 36–38] and nine evaluated reminders by text [26], telephone call [26, 31, 33, 37] and/or letter [25, 29, 32–34, 37, 38], including two using letters with timed appointments [25, 32]. Two studies included an educational component as part of the interventions [27, 33], and two addressed access barriers [27, 31]. Another two studies assessed the impact of different screening modalities: one assessed the impact of letters for a range of tests offered at different frequencies [28], and the other evaluated organised HPV testing at different coverage rates [36].

In countries where there is an established organised screening programme, studies used the standard invitation as the comparator following the screening practices in that particular country context [22–26, 28–31, 34, 36–38]. Two studies used no screening [24, 27] and three used opportunistic screening as the comparator [32, 33, 35].

### Cost-effectiveness of interventions to increase uptake rates

#### Self-sampling

Four studies conducted cost-utility analyses of self-sampling to increase the uptake rates of cervical cancer screening, using QALY as health outcomes [22–25]. Three of these reported ICERs ranging from €2,377/QALY to €26,446/QALY and concluded that self-sampling as an add-on to standard screening was cost-effective against the Norwegian, Dutch, and UK thresholds, respectively [22, 23, 25]. Vassilakos et al. evaluated self-sampling and triage with cytology, self-sampling, and triage with colposcopy versus standard strategy (cytology and triage with HPV) in Switzerland. The results showed that self-sampling was found to be more efficient and cost-saving than the standard strategy, and self-sampling with triage by cytology was found to be the most cost-effective strategy in underscreened women [24].

Four cost-effectiveness studies assessed self-sampling using cost per screen [29] or cost per CIN2 + [36–38] as the outcomes. The ICERs were €69 per additional screen or €4784–€11,825 per additional CIN2 + and the studies concluded that self-sampling was preferred and effective without markedly increasing the costs [29, 36–38].

#### Reminder interventions

Reminder interventions have been explored to increase cervical cancer screening uptake rates, including text, telephone calls, and/or letters.

Firmino-Machado et al. found that text messages and automated phone calls were cost-saving compared to the standard invitation involving written letters from the women’s registered primary care unit [26]. Additionally, the use of text messages, automated phones, and manual calls was cost-saving from the healthcare perspective and cost-effective from the societal perspective. The study also evaluated, as another arm, the addition of a face-to-face appointment for those that did not respond to text messages, automated calls and manual calls: this had an ICER of €633/QALY from the healthcare perspective and €6,250/QALY from the societal perspective, well below the stated cost-effectiveness threshold [26]. Tsiachristas et al. showed that a letter with a timed appointment for cytology was cost-effective with an ICER of €11,634/QALY, as well as a letter offering women the choice of either having access to a nurse navigator or a requested HPV self-sampling kit being cost-effective with an ICER of €10,882/QALY [25].

Overall, the cost-effectiveness studies found that the ICERs of reminder letters from health professionals ranged from €40 to €85 per additional woman screened [29, 30, 34] or €18,058 per additional CIN2 + [38]. Reminder letters with timed appointments had an ICER of €11 per additional woman screened, which rises to €15 when coupled with another reminder letter in the context of only opportunistic screening in Lithuania [32]. Telephone reminders were reported as being cost-effective compared to standard invitation in Sweden, with an ICER of €4420 per additional CIN2 + treated [37]. However, in the UK, telephone reminders were dominated by letter invitations from a healthcare professional [30].

#### Multicomponent interventions to improve access

Voko et al. reported that the addition of greater awareness raising (e.g. increased presence on mass media, letters, information leaflets, involvement of local opinion leaders, and general practitioners) to current screening programmes (based on combined cytology and colposcopy in gynaecological outpatient services) had an ICER of €39,145/QALY [27]. An alternative scenario with the same awareness raising measures but using trained public health nurses to undertake Pap smears only general practitioner offices, and thus closer to women’s homes, had an ICER of €22,458/QALY [27]. Both interventions were concluded to be cost-effective compared with the existing service, which required attendance at gynaecology outpatient clinics [27]. By contrast, Oscarsson et al. evaluated an intervention consisting of a telephone call and personalised practical arrangements, found an ICER of €202 per additional woman screened [31]. Trapero-Bertran et al. reported that invitation letters, leaflets, and telephone calls incurred a cost of €61 per additional 1% screening coverage [33].

#### Organised screening programme

In countries where existing screening is carried out on opportunistic basis, an intervention to improve screening may be the introduction of an organised screening programme. The registry source from which eligible women are identified, age range, type of primary and confirmatory tests used, and the frequency of testing across a woman’s lifetime vary. Barré et al. assessed organised cervical cancer screening strategies at varying time intervals, with varying primary and confirmatory tests, compared with opportunistic screening. They concluded that organised screening strategies based on HPV testing appear cost-effective, but the authors acknowledged that feasibility may determine the choice of screening tests used [28]. Diaz et al. compared the current policy of opportunistic cytology screening with a modelled organised programme based on primary HPV screening and concluded that organised screening would provide greater coverage for the same total costs [35].

### Methodological assessment

The methodological assessment of the reviewed studies were summarised in Table 2. Nine studies were model-based analyses, with two using decision tree models [26, 32], three using Markov models [25, 27, 35], one using both decision tree and Markov models [24], and three using microsimulation models [22, 23, 28]. The other eight studies were trial-based economic evaluations [29–31, 33, 34, 36–38].

**Table 2:** Methods used in the included papers

Study	Economic analysis approach	Cost perspective	Time horizon	Discount rate	Sensitivity analysis
Burger et al. [22]	Microsimulation	Societal	Lifetime	4%	DSA and PSA
Rozemeijer et al. [23]	Microsimulation	Societal	Lifetime	3%	DSA
Vassilakos et al. [24]	Decision tree and Markov	Healthcare	Lifetime	3%	DSA and PSA
Tsiachristas et al. [25]	Markov	Healthcare	Lifetime	3.5%	DSA and PSA
Firmino-Machado et al. [26]	Decision tree	Healthcare and societal	5 years	3%	DSA
Voko et al. [27]	Markov	Healthcare	20 years	5%	DSA and PSA
Haguenoer et al. [29]	Trial based	Societal	1 screening cycle	n/a	DSA
Bais et al. [36]	Trial based	Societal	1 screening cycle	n/a	None
Broberg et al. [37]	Trial based	Healthcare	1 screening cycle	n/a	None
Virtanen et al. [38]	Trial based	Healthcare	5 years	n/a	None
Stein et al. [30]	Trial based	Healthcare	1 screening cycle	n/a	None
Oscarsson et al. [31]	Trial based	Healthcare	1 screening cycle	n/a	None
Paulauskiene et al. [32]	Decision Tree	Healthcare	1 screening cycle	5%	DSA
Trapero-Bertran et al. [33]	Trial based	Healthcare	3–5 years	n/a	DSA
Barré et al. [28]	Microsimulation	Societal?	Lifetime	4%	DSA
De Jonge et al. [34]	Trial based	Healthcare	3 years	n/a	None
Diaz et al. [35]	Markov	Societal	Lifetime	3%	DSA

Six studies took a societal perspective [22, 23, 28, 29, 35, 36], ten studies took a healthcare perspective [24, 25, 27, 30–34, 37, 38], and the remaining study reported results from both societal and healthcare system perspectives [26]. Six studies considered costs and health outcomes over a lifetime horizon [22–25, 28, 35], and others specified a time horizon varying in length between 3 years and 20 years [26, 27, 33, 34, 38], or defaulted to one screening cycle [29–32, 36, 37]. Nine studies applied discounting rates between 3 and 5% to future costs and benefits [22–28, 32, 35]. Eleven studies explored uncertainty in their results through deterministic sensitivity analysis [22–29, 32, 33, 35] or probabilistic sensitivity analyses [22, 24, 25, 27].

### Critical appraisal of study quality

The included studies are of variable quality which is presented in Table 3. Evidence of effectiveness of interventions often relied on single trials. In the single study that synthesised effectiveness results from multiple trials, there was no comment on the weighting of results based on the quality of evidence [24]. Sources of bias noted in trials included post hoc changes to intervention design prompted by unexpectedly low response rates [37], incomplete information on randomisation [36], intervention by unblinded lead researcher [31], differential treatment of intervention and control groups [22], incomplete data collection [34], and participation bias incurred through exclusion of non-responders [37].

**Table 3:**
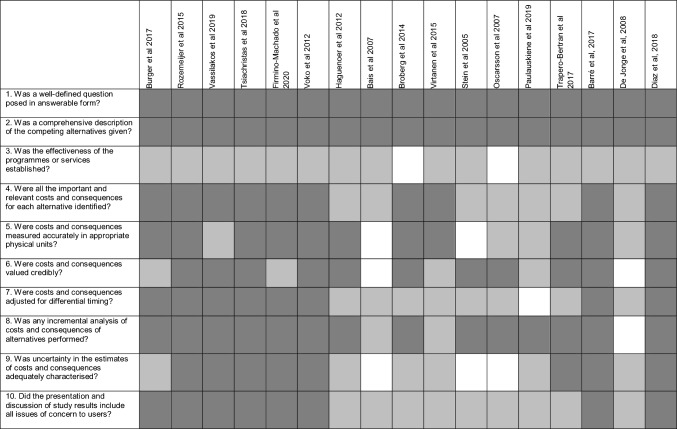
Critical appraisal

Key Fully Met  Partially Met  Absent

Taken as a whole, the studies systematically described costs, consequences, and their derivation. However, four studies failed to consider the costs associated with follow-up and treatment of abnormalities detected through increased screening participation [29, 32–34]. The studies that used a longer time horizon employed discounting, but gave varying degrees of justification for the choice of discount rate applied. Only two studies considered that after an intervention, there may be variable adherence in subsequent screening cycles, with the rest assuming lifetime improved uptake [25, 35].

The quality and depth of presentation and discussion of study results varied. Overall, the conclusions drawn from the ICERs calculated were interpreted within the local context and comparisons were drawn to existing evidence. In exploring the uncertainty around cost-effectiveness estimates, only four of the eleven studies that conducted some form of sensitivity or scenario analysis, undertook a probabilistic sensitivity analysis [22, 24, 25, 27]. This provided more useful information for policy makers by supplying probability data as to whether the intervention was cost-effective across a range of thresholds and allowed simultaneous assessment of multiple strategies.

Generalisability was, for the most part, considered to be limited to within the country context of each study. This is inevitable as the disadvantage experienced by a particular group is likely to reflect a wide range of cultural, historical, and legal factors. The most pertinent factors discussed, that affect wider generalisability, relate to the presence or absence of an organised screening programme and/or screening registry [22, 24, 28, 32, 34, 37, 38], prevalence of hrHPV in the population [22, 23], as well as out of pocket expenditure for women associated with screening [23]. Ten of the studies considered factors other than cost-effectiveness that might influence whether the intervention should be adopted. These included presence and coverage of HPV vaccination programmes [23, 25]; the ability of the intervention to reach those most at risk [23, 27, 33, 37] and the potential for overscreening [22, 28].

## Discussion

### Summary of findings

This study systematically reviewed published studies on the cost-effectiveness of interventions to increase cervical cancer screening uptake rates in underserved women in Europe.

Self-sampling and reminder interventions were generally shown to be cost-effective to increase uptake rates among underscreened women. There are a limited number of studies showing that addressing access issues and adopting different screening modalities could be economically attractive, but the evidence is limited.

Another key finding is that the existing evidence base does not take account of intersectionality or of policy-relevant distinctions within groups of underserved women, such as certain migrant groups or racially minoritised communities. All the included studies evaluated interventions in underscreened women as an overarching group. Twelve subgroups of women were identified as underserved as part of the search strategy, with some women belonging to multiple subgroups. However, no economic evaluations were identified that focused on cost-effectiveness of an intervention in any particular sub-group. Overall, the majority of included studies conclude that interventions to increase uptake of cervical cancer screening among underscreened women are cost-effective, although this was not always discussed in reference to formal willingness-to-pay thresholds.

### Limitations of the reviewed studies and need for further research

#### Understanding who is underserved by existing screening in Europe

The lack of economic evidence for interventions aimed at specific underserved groups is compounded by the lack of sub-group analysis of who is responsive to interventions aimed at all underscreened groups. This results in a gap in our understanding of who is reached by population-level interventions, and represents a missed opportunity to reduce health inequalities. The need for targeted interventions may seem at odds with a population-based screening programme aiming for universal coverage, although it is consistent with the concept of progressive universalism, whereby a service is available to all but measures are taken to eliminate barriers that arise out with it [39]. However, given the existing pattern of inequalities in the burden of cervical cancer, the literature insufficiently describes how a universal offer meets the needs of those with the highest morbidity and mortality from the disease. In fact, studies have excluded underserved groups from trials aimed at all underscreened women. For example, Stein et al. excluded women with disabilities from their study [30]. It is important that unintended segregation is avoided in the tailoring of services to specific groups, e.g. using patient contacts with the health service for other health reasons could create an opportunity for screening, outside the usual channels of the organised screening, without this concern of segregation.

The definition of underscreened varies according to the recommended screening interval in a particular country context. Only a single study accounted for screening history in its methodology, incorporating the assumption that women who were least responsive to standard screening offer, were also likely to be least responsive to the intervention in their model [22]. A large study of more than 55,000 women in Belgium and Switzerland revealed how determinants of screening inequalities differ among never- and under-screeners. Of note, they reported socio-economic and demographic inequalities were more pronounced among never-screeners who appeared to face more structural and persistent inequalities [40].

#### Building the evidence base: methodological challenges

There is considerable heterogeneity in the economic evidence base relating to the cost-effectiveness of interventions to increase screening uptake amongst underscreened women, in terms of both economic evaluation methods and study designs. Notable gaps in methodology relate to assumptions around screening coverage and compliance and not accounting for HPV vaccination rates.

The predominance of trial-based evaluations skews the evidence base to shorter time horizons, usually the length of the trial or one screening cycle representing 3–5 years. This is problematic due to the long natural history of cervical cancer and the need for repeated screening tests over 30–40 years. Future costs and benefits are not adequately captured in these analyses. This is compounded by the strong assumption made in all but one study [25] that responsiveness to an intervention in one screening cycle will result in a lifetime of compliance with future screening practices (although this assumption is not explicitly stated in the methodology of the majority of studies). Lifetime compliance after a one-off intervention might not necessarily occur, for example, if the theory of change for an intervention relies on a behavioural nudge (e.g. reminder from primary care physician) or removing access issues (e.g. self-sampling), these will likely need to be repeated in future screening cycles, and therefore costed into models assessing cost-effectiveness. This notion is supported by the similar effectiveness of interventions on both underscreened and never screened populations, indicating that previous participation in screening is not an accurate predictor of future compliance across a screening lifetime of 20–30 years.

None of the included studies accounted for HPV vaccination in the base case cost-effectiveness analyses. Since 2006, HPV vaccination has been offered in many countries, with European coverage rates for the final dose ranging from 14 to 83% [19, 41]. There has been recent evidence stipulating that vaccinated women still require either 2–3 screens for cervical cancer during their lifetime [42]. Thus screening needs in a country will vary based on the vaccination coverage rates, while policies must also account for the burden of disease caused by types that are not vaccine preventable.

This review focuses on uptake rates for initial screening tests; however, questions around cost-effectiveness of such interventions need to also account for subsequent participation in diagnostic testing and treatment in order to fully understand the cost-effectiveness across the screening pathway. Whilst some authors acknowledge the potential for loss to follow-up, this was rarely accounted for in base case cost-effectiveness determinations. An assumption was made regarding adherence to subsequent stages of the screening pathway, possibly resulting in an overestimation of benefits of increasing participation in the initial diagnostic stage.

### Strengths and contributions of this review

A key strength of our review was undertaking double-blinded screening and data extraction to minimise the risk of bias within individual reviewers. Another strength is that no language limits were applied to the search strategy.

The findings of this review add to the existing evidence base suggesting that interventions that are effective in improving participation in cervical cancer screening, can also be cost-effective. Previous systematic reviews have looked at the inequalities in the uptake of screening [43–45], barriers to cervical cancer screening [46, 47], as well as efficacy of interventions to increase uptake [18, 48]. This review advances our understanding of the cost-effectiveness of interventions to improve uptake of cervical cancer screening in underserved women and highlights areas for further research as outlined above.

### Policy/programmatic recommendations/implications

Implementing self-sampling and reminder interventions can be cost-effective for increasing uptake rates among underscreened women but should be accompanied by adequate monitoring of uptake among subgroups of underserved women.

When interventions to improve cervical cancer screening are implemented, in addition to uptake rates of screening, there should be active monitoring of loss to follow-up across the screening pathway among subgroups of underserved women. Key indicators include attendance rates for diagnostic testing and treatment after a positive screening and diagnostic result, respectively. Currently, we are working on the CBIG-SCREEN EU funded project that will evaluate the cost-effectiveness of co-created interventions to increase screening in different vulnerable groups [49].

## Conclusion

Self-HPV testing and reminder interventions were generally shown to be cost-effective to increase uptake rates among underscreened women. There are a limited number of studies showing that addressing access issues and adopting different screening modalities could be economically attractive. This systematic review has revealed a gap in the literature on the cost-effectiveness of interventions to improve uptake rates of cervical cancer screening among underserved women in Europe. The factors determining the risk profile for being susceptible to HPV, as well as the barriers and facilitators of screening, are specific to each different group. Targeted interventions aiming to redress these, need to be evaluated in terms of their cost-effectiveness. If interventions are aimed at all underscreened women, sub-group analysis should be conducted to describe the reach of these interventions and their impact on specific populations.

Future economic evaluations of interventions to increase cervical cancer screening participation should have an explicit focus on underserved women and different subgroups within this overarching group, as well as taking into consideration HPV vaccination coverage and adherence across screening cycles.

### Supplementary Information

Below is the link to the electronic supplementary material.

Supplementary file1 (DOCX 15 KB)

## Data Availability

This is a systematic literature review and all summary data will be reported in the manuscript and could be used by other researchers.

## Footnotes

1 Throughout this review, it is intended to be inclusive of all those who are at risk of developing cervical cancer, i.e. all individuals with a cervix, which includes transgender men. Any reference to “women” is not intended to exclude transgender men; rather it is a reflection of the prevailing terminology in the current literature base.
