# Supplementary material for: A systematic review of the cost-effectiveness of interventions to increase cervical cancer screening among underserved women in Europe
Source: Eur J Health Econ. 2023 Sep 20;25(5):829–44. doi: 10.1007/s10198-023-01627-1 (PMC11192698; doi:10.1007/s10198-023-01627-1)
Supplement: Supplementary file 1 — Supplementary file1 (DOCX 15 KB) [file 10198_2023_1627_MOESM1_ESM.docx]

# Appendix

Example Search Strategy : Embase

1. exp uterine cervix cancer/ or exp uterine cervix carcinoma/

2. exp Papanicolaou test/ or exp cancer screening/ or exp mass screening/

3. 1 and 2

4. ((Cervical or Pap or Papanicolaou or HPV or "high-risk HPV" or hrHPV or "Human Papillomavirus" or smear) adj5 (screen* or test* or smear)).mp.

5. 3 or 4

6. exp economic evaluation/

7. ((Economic* or costing or cost or costs or costed or financ*) adj5 (evaluat* or minimis* or effective* or analys*or benefit* or utility or consequence)).mp.

8. economic model*.ab.

9. markov.ti,ab.

10. monte carlo method/

11. 6 or 7 or 8 or 9 or 10

12. 5 and 11

13. exp Europe/

14. (europe* or EU or EEA).mp.

15. (Albania or Andorra or Armenia or Austria or Azerbaijan or Belarus or Belgium or "Bosnia and Herzegovina" or Bulgaria or Croatia or Cyprus or "Czech Republic" or Denmark or Estonia or Finland or France or Georgia or Germany or Greece or Hungary or Iceland or Ireland or Italy or Kazakhstan or Kosovo or Latvia or Liechtenstein or Lithuania or Luxembourg or Macedonia or Malta or Moldova or Monaco or Montenegro or Netherlands or Holland or Norway or Poland or Portugal or Romania or Russia or "San Marino" or Serbia or Slovakia or Slovenia or Spain or Sweden or Switzerland or Turkey or Ukraine or "United Kingdom" or England or Wales or Scotland or "Northern Ireland" or "Great Britain" or "Vatican City").mp.

16. 13 or 14 or 15

17. 12 and 16
